# An outbreak of Panton-Valentine leucocidin-producing meticillin-resistant *Staphylococcus aureus* skin and soft tissue infection associated with bouncy castle use, Ireland, 2025

**DOI:** 10.2807/1560-7917.ES.2026.31.36.2500975

**Published:** 2026-09-10

**Authors:** Domhnall McGlacken-Byrne, Gráinne Brennan, Eimear Kitt, Keith Ian Quintyne, Eimear Brannigan, Máirín Boland, Andrea King, Michael Barrett, Noelle O’Loughlin, Desmond Hickey

**Affiliations:** 1 Department of Public Health, Dublin and Midlands, Health Service Executive, Dublin, Ireland; 2 National MRSA Reference Laboratory, Dublin, Ireland; 3 Children’s Health Ireland at Crumlin, Dublin, Ireland; 4 National Health Protection Office, Health Service Executive, Dublin, Ireland; 5 Antimicrobial Resistance and Infection Control Team, Office of the Chief Clinical Officer, Health Service Executive, Dublin, Ireland

**Keywords:** Meticillin-resistant *Staphylococcus aureus* (MRSA), Panton-Valentine leucocidin (PVL), Skin and soft tissue infection, Community MRSA, Public health, Health protection

## Abstract

We describe an outbreak of Panton-Valentine leucocidin (PVL) producing community-acquired meticillin-resistant *Staphylococcus aureus* (CA-MRSA) in children and adolescents in eastern Ireland, associated with bouncy castle use. Regional public health authorities were notified in autumn 2025 of several hospital presentations by children and adolescents with skin and soft tissue infection (SSTI). An outbreak was declared. Phone interviews were conducted with a parent or guardian of each case, obtaining demographic, clinical and exposure information. All 48 identified cases were children and adolescents. Of these, 47 attended the same outdoor social gathering in autumn 2025 which featured bouncy castle play. Case definitions were drafted and iteratively refined. The commonest affected body site was the legs, particularly thighs. Thirty-three cases were treated by their general practitioner (GP). A further 15 attended a hospital emergency department or day ward, of whom four were hospitalised, with no deaths. All confirmed cases exhibited an identical strain type, t016-ST22-MRSA-IV, which was PVL-producing. This strain has been described as hypervirulent but is rare in Ireland. The causal isolate exhibited a distinctive antibiogram. Key control measures for this complex outbreak included early recognition of linked cases, rapid delivery of clinical care, and infection prevention and control measures.

**Figure:**
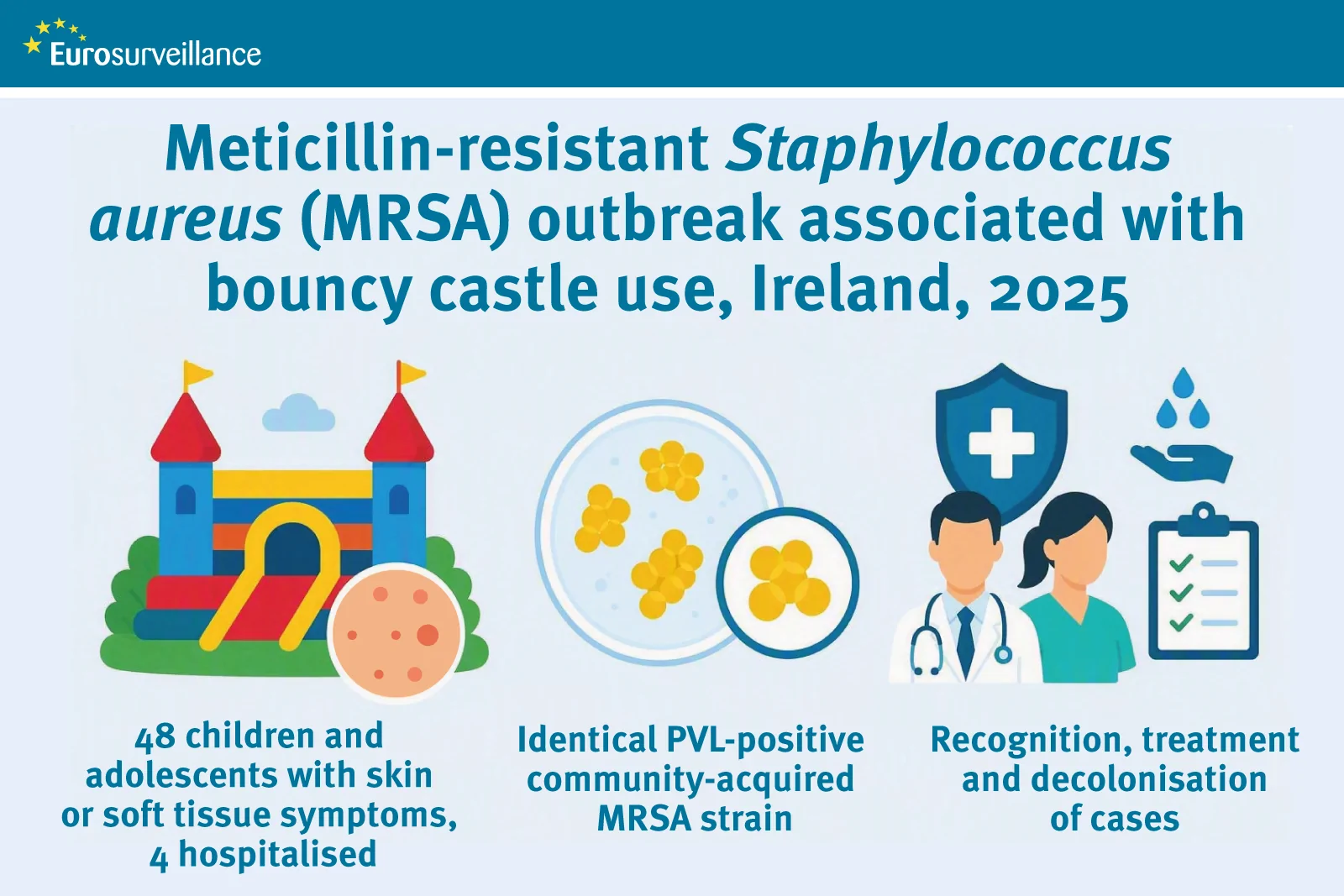

**Key public health messages**

**What did you want to address in this study and why?**

Meticillin-resistant *Staphylococcus aureus*, or MRSA, can cause skin and deep tissue infections difficult to treat. In this report, we describe the measures taken to control a large and complex outbreak of MRSA, among children and adolescents in eastern Ireland, associated with bouncy castle use.

**What have we learnt from this study?**

This outbreak consisted of 48 cases of skin and soft tissue infection, including four hospitalised children, which is the largest MRSA outbreak on record in Ireland. Control of the outbreak was achieved by a combination of control measures, including treatment of cases, decolonisation treatment of household contacts, and communication with stakeholders such as the bouncy castle providers.

**What are the implications of your findings for public health?**

We consider that the outbreak occurred due to close physical contact among children and adolescents playing on bouncy castles. This mechanism, in an outbreak of this size, has not been described before in the scientific literature. A multidisciplinary outbreak control team was needed in the outbreak investigation and response.

## Background

Community-acquired meticillin-resistant *Staphylococcus aureus* (CA-MRSA) outbreaks are associated with close physical contact and crowded settings, such as sporting activities [1-6], prisons [7] and childcare facilities [8,9]. Evidence from several outbreaks suggests that skin barrier breakage, for instance due to burns or abrasions, is a potential propagating factor [1,3,4,10]. The potential role of inflatable play structures, like bouncy castles, in CA-MRSA transmission has not been documented in peer-reviewed literature.

## Outbreak detection

The Department of Public Health for the Health Service Executive Dublin and Midlands region was notified in autumn 2025 of a 12-year-old male patient with MRSA folliculitis, by a member of the paediatric infectious diseases team of a tertiary children’s hospital. The patient, who was hospitalised, provided a history of recent attendance at a social gathering in eastern Ireland, subsequent to which ca 25 fellow attendees reportedly also developed symptoms of skin and soft tissue infection (SSTI). This gathering had occurred 9 days previously. Paediatric clinicians observed a plausible epidemiological link between early cases of SSTI, presenting with similar histories and clinical findings, which led to the initial signal notification to public health.

Here we present the investigation and management of this outbreak of CA-MRSA skin and SSTI in children and adolescents in autumn 2025, linked to close contact play involving use of bouncy castles and other inflatable play structures.

## Methods

An outbreak control team (OCT) was urgently convened, with representation from regional and national public health authorities, paediatric clinicians, clinical microbiology, national antimicrobial stewardship and infection control, and the regional environmental health service.

### Case definition

A case definition was drafted and iteratively refined, comprising possible, probable and confirmed cases (Box).

Box **Definitions of possible, probable and confirmed cases with meticillin-resistant *Staphylococcus aureus* (MRSA), Ireland, 2025**

**Possible case:**

Clinical and epidemiological criteria:

• A person with onset of symptoms of skin and soft tissue infection or a clinically compatible presentation such as bacteraemia or pneumonia after the date of the event, who attended the gathering or had an epidemiological link to it.

AND

Laboratory criteria:

• No laboratory testing or no MRSA detected.

**Probable case:**

Clinical and epidemiological criteria:

• As for a possible case

AND

Laboratory criteria:

• MRSA detected from a clinical specimen, the isolate exhibited a characteristic antibiogram profile: resistant to flucloxacillin, ciprofloxacin, gentamicin and trimethoprim, and susceptible to clindamycin, tetracycline, fusidic acid, co-trimoxazole and linezolid.

**Confirmed case:**

Clinical and epidemiological criteria:

• As for a possible case

AND

Laboratory criteria:

• MRSA detected from a clinical specimen, the isolate characterised with whole genome sequencing (WGS) as strain t016-ST22-MRSA-IV, showing ≤ 24 allelic differences.

ST: sequence type.

### Case detection

Initial communications and information-gathering were primarily between the regional department of public health, paediatric clinicians, local general practitioners (GPs) and families who had attended the event. As more cases were notified, a parent or guardian of each case was interviewed by phone by a medical or nursing member of the department’s health protection team. A line list document was rapidly compiled and populated, comprising ca 30 variables pertinent to the demographic, clinical and exposure-related characteristics of each case.

Outbreak cases were actively sought and accounted for by several methods. Firstly, each time a case was reported by acute services, a public health risk assessment was conducted, in line with the departmental standard operating procedure (SOP). Contact with the party host enabled identifying families who had attended this party. Each family was individually contacted by a member of the public health team, and a risk assessment was conducted to determine if any had developed symptoms of skin infection. Contact details for other cases were sought purposively. Families of these suspected cases were then directly contacted by a member of the public health team. Lastly, the public health team liaised closely with the regional and national microbiology laboratories, to monitor for incoming MRSA isolates matching the outbreak profile.

### Microbiological investigations

Skin swabs were collected from site infections on patients and submitted to local microbiology laboratories. Presumptive MRSA isolates recovered from samples were referred to the National MRSA Reference Laboratory, where they were confirmed as MRSA and underwent spa typing and whole genome sequencing (WGS) using the Illumina DNA Prep kit and MiSeq platform (Illumina, San Diego, the United States (US)) as previously described [11].

## Results

### Epidemiological investigation

#### Case descriptions

**Figure 1:**
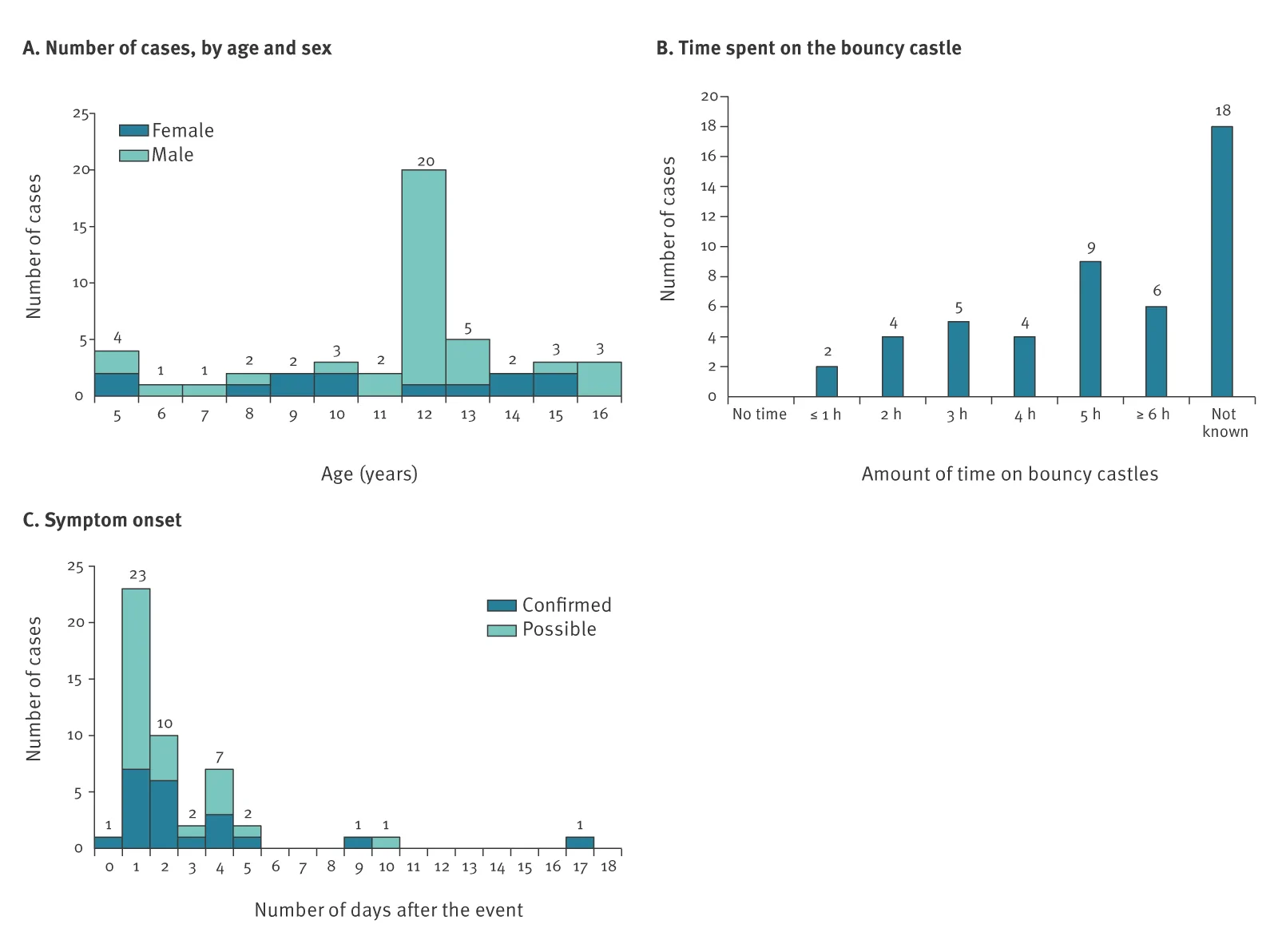
Timeline of outbreak of meticillin-resistant *Staphylococcus aureus* (MRSA) associated with bouncy castle use, by age, sex and exposure time, Ireland, 2025 (n = 48)

This outbreak comprised 48 cases: 26 possible and 22 confirmed but no probable cases. There were no adults among the cases. The case ages ranged from 5 to 16 years (median: 12 years) (Figure 1A). Most cases were male (35/48) (Table).

**Table:** Selected characteristics of cases with meticillin-resistant *Staphylococcus aureus* (MRSA) in an outbreak associated with bouncy castle use, Ireland, 2025 (n = 48)

Characteristic	Number	%
Age (years)		
Median	12	
Interquartile range	10–13	
Sex		
Female	13	27
Male	35	73
Symptom type		
Skin symptoms only	36	75
Skin symptoms and fever	5	10
Skin symptoms and nausea or vomiting	7	15
Body site location^a, b^		
Legs	20	42
Abdomen or trunk	12	25
Hips or buttocks	6	13
Arms or hands	6	13
Head or neck	1	2
Body site not specified	21	44
Healthcare attendance type		
General practitioner only	33	69
Emergency department or day ward only	11	23
Emergency department plus admission	4	8

^a^ Body site locations are not mutually exclusive. As such, the subtotals in these rows sum to more than 48.

^b ^For 27 cases, the body site location of symptoms was successfully documented.

All cases reported skin or soft tissue symptoms (Table). These were variably reported as spots, pustules, redness, blisters, abscess formation, or other descriptive skin and soft tissue-related terms. Skin lesion site distribution was not systematically collected but was documented where possible: in 21 cases, body site distribution was not documented, while it was documented for the remaining 27 cases. Among cases where body site was documented, the commonest body location reported was the legs (20/27) – in particular the thighs – followed by the abdomen or trunk (12/27), the hips or buttocks (6/27), the arms or hands (6/27), and the head and neck (1/27) (Table). Some cases had symptoms affecting multiple body parts. In addition, 12 cases reported systemic symptoms, such as fever, nausea or vomiting. There were no deaths associated with this outbreak.

All cases sought medical attention: 33 from the GP and 15 from hospital. Four cases were admitted to hospital, though none had severe outcomes or required intensive care. The median duration of hospitalisation was 4 days. Cases who attended hospital were more likely to have had a swab taken and so to be classified as confirmed cases. Of the 15 cases who attended hospital, 12 were confirmed and 3 were possible cases. Of the 33 cases who were managed by their GP, 10 were confirmed and 23 were possible cases. Of the 26 possible cases, no swab was reported as having been taken for 21 of them, while in five cases a skin swab was taken which yielded no growth. Cases received treatment including antibiotics as clinically appropriate and decolonisation. Decolonisation consisted of a chlorhexidine-based or equivalent antimicrobial body wash, applied once daily for 5 days, along with mupirocin nasal antimicrobial ointment for 5 days.

All cases developed symptoms subsequent to the gathering in question, including one case who became symptomatic within hours of attendance (Figure 1C). Most cases (34/48) developed symptoms within 2 days of the gathering. One confirmed case, who attended the social gathering, developed symptoms 17 days following the event.

### Event description

Information-gathering efforts revealed that the social event in question took place on a single afternoon and evening on a green area in a suburban community. It was a single event with no related events. The day was described as humid, warm and intermittently rainy. Parents recalled that children and adolescents playing outside were noticeably wet. There was no definitive list of attendees for the event, with invitation occurring informally. It was estimated that ca 120 individuals attended, of whom 60 were children and adolescents. The gathering included an outdoor gazebo, a barbecue, a confectionary station and a range of inflatable play structures including three bouncy castles. The bouncy castles were provided expressly for the event and removed afterwards.

All but one patient had attended the social event (47/48). One possible case with symptom onset 3 days after the event, but who did not attend, was in sustained social contact with another child who was reported to have attended the event and who also had SSTI symptoms. Of the 47 cases identified, who attended the social gathering, all played on the bouncy castles. As shown in Figure 1B, it was apparent that most children and adolescents spent substantial time on the bouncy castle – for example, 19 of 47 cases were recalled as having spent ≥ 4 h playing on the bouncy castle.

Parents were asked about other exposures which may have led or contributed to the outbreak, in the days and weeks before the social gathering. Ten cases were reported as having participated in the same group sport activity in the preceding week.

There were seven families in whom more than one member developed symptoms; all had attended the party. At the time of outbreak closure, there were also four reports of known cases who subsequently experienced symptomatic infection recurrence. The outbreak was declared closed once two maximum incubation periods had lapsed since the last new case. This was 40 days after the social gathering in question and 31 days after the initial notification to the public health department.

### Microbiological results

**Figure 2:**
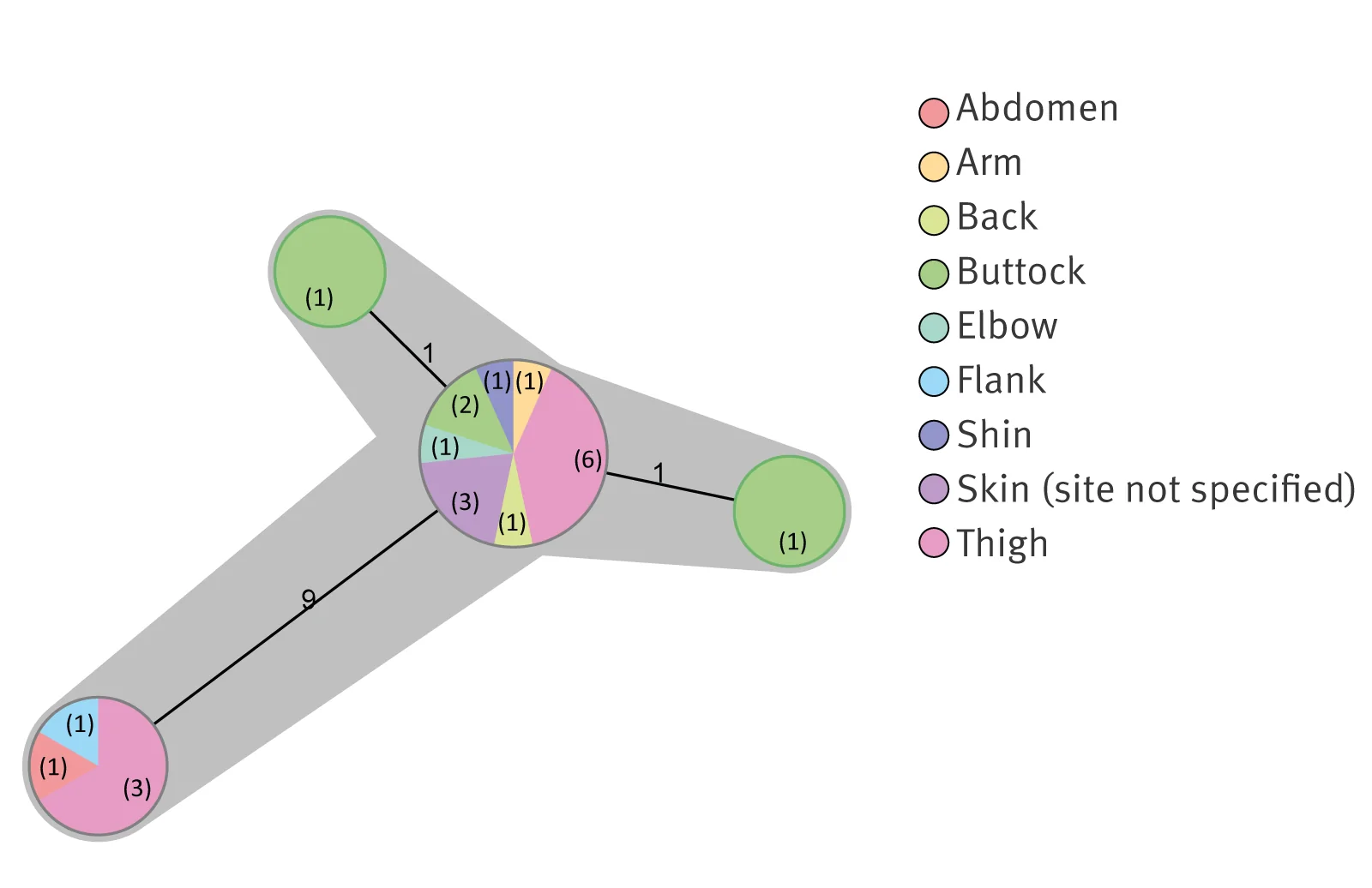
Minimum spanning tree based on core genome multilocus sequence typing (cgMLST) for isolates of meticillin-resistant *Staphylococcus aureus* from confirmed cases in an outbreak associated with bouncy castle use, Ireland, 2025 (n = 22)^a^

^a^ A cluster distance threshold of 24 allelic differences was used.

In the confirmed cases, WGS identified an MRSA strain, ST22-MRSA-IV, and spa type t016. All samples were of staphylococcal cassette chromosome (SCC)*mec* type IV. All isolates were Panton-Valentine leucocidin (PVL) producing. A minimum spanning tree based on 1,861 genes was generated and showed that all isolates clustered together in a single cluster differing by a maximum of nine allelic differences (Figure 2). By convention, isolates exhibiting 24 or fewer allelic differences are considered closely related [12-14]. With respect to virulence gene content, the only relevant gene identified was the PVL gene.

Minimum spanning tree was generated using Ridom Seqsphere + version 10.0.6 (https://www.ridom.de/seqsphere/ug/v40/Overview.html). Isolates are categorised based on the sampling site with the number of isolates in each category shown on the tree in parentheses. All isolates are grouped into four separate nodes with the number of allelic differences between nodes shown above the black lines.

## Outbreak control measures

Communication and public health advice was a central strategy in managing this dynamic and complex outbreak.

In the interviews with parents or guardians, advice on appropriate control measures was conveyed. Prior to the phone interviews, general information on the outbreak and a link to key infection prevention and control (IPC) advice was also conveyed by text message to support rapid communication. Paediatric and public health OCT members collaborated in standardising advice to cases’ families. Furthermore, detailed information was sent by the public health department, via a messaging platform, also for those who attended the party but were asymptomatic. This communication is included in Supplement.

An alert was sent by the regional public health department to local hospitals, for cascading to relevant clinical colleagues within their services, flagging the outbreak and the distinctive antibiogram of the attendant strain. The local GPs and pharmacy were provided with informational materials in support of response to potential queries from cases, contacts or members of the public. The local pharmacist assisted with the logistics associated with the sudden increase in local demand for disinfectant products.

The regional public health department contacted each of the equipment providers at the gathering, including the catering and gazebo companies and the play equipment company which provided the bouncy castles and other inflatable play structures. Advice was issued to these third parties that the inflatable play equipment and associated transport should not be reused at other events until adequately cleaned and disinfected. The public health team recommended that this cleaning should include removal of visible soilage followed by cleaning with disinfectant cleaning wipes of all reachable surfaces. The play equipment company stated that, previous to this outbreak, inflatable play equipment underwent routine systematic cleaning only when visibly soiled after uses.

The local primary school, attended by the majority of cases, was notified of the outbreak and was issued advice on infection prevention and control measures, the need for cases’ temporary exclusion from school and guidance in case of a child developing symptoms. A local sports club was also contacted and provided with advice.

Senior regional health service leadership, national health protection leadership and the Department of Health were updated on the outbreak. A notification was published on EpiPulse, an online portal operated by the European Centre for Disease Prevention and Control (ECDC).

The multi-disciplinary OCT met on five occasions in the 4-week period after notification. The outbreak was declared over following the lapse of two maximum incubation periods (20 days) since the last new case.

## Discussion

This report describes the investigation and control of a large cluster of SSTIs associated with PVL-producing CA-MRSA in children and adolescents who attended the same social gathering in eastern Ireland in autumn 2025. Of 48 cases, 22 were confirmed on WGS as belonging to the same MRSA strain – ST22-MRSA-IV and *spa* type t016 – while 26 were classified as possible. This represents the largest reported CA-MRSA outbreak that has occurred in Ireland (personal communication: F Cloak, Health Protection Surveillance Centre (HPSC), October 2025).

The outbreak investigation and response were resource-intensive and required close collaboration among multiple stakeholders, including regional and national public health teams, antimicrobial stewardship and infection control experts, the national reference laboratory, paediatric clinicians and environmental health colleagues.

While ST22-MRSA-IV is widely reported as a healthcare-associated MRSA (HA-MRSA) lineage throughout Europe, accounting for as much as 80% of MRSA recovered from bloodstream infections in Ireland in previous years [15,16], this strain is distinct to the previously reported HA-MRSA strains. The t016-ST22-MRSA-IV lineage produces PVL and harbours multiple resistance genes encoding resistance to flucloxacillin, ciprofloxacin, gentamicin and trimethoprim. Previously, PVL-positive ST22-MRSA-IV has been linked to outbreaks in India, Japan and China [17-19]. Cases in Ireland are usually sporadic, where this strain has not been previously linked to an outbreak in either a healthcare setting or the community [20]. There was only one prior instance of a t016-ST22-MRSA-IV lineage isolated by the Irish National MRSA Reference Laboratory, in eastern Ireland, some years previously.

This outbreak is also notable for its complexity and apparently high attack rate. It was not possible to capture a definitive list of attendees at the gathering, but estimates stood at 120, of whom approximately half were children and adolescents. This suggests an attack rate of ca 80%. In addition, the incubation time between the presumed exposure at the gathering and symptom onset in most cases was relatively short. Evidence on the incubation time of MRSA in the context of SSTI is scant, but it has been described as 4–10 days [21]. Incubation time may be shortened where the inoculum is high, skin abrasions are present, or the requisite strain is highly virulent [1,2,18]. In this outbreak, most cases developed symptoms in fewer than 4 days, and in multiple cases within 24 h.

The OCT considered various hypotheses to explain this outbreak. The most apparent epidemiological link was that every case attended the same party – except for one case, who did not attend but had reported contact with an attendee who may have had symptoms of SSTI subsequent to attending the event. No cases were reported in adults, suggesting a differential exposure at the party among children and adolescents.

The OCT also considered various approaches to testing cases and contacts post-decolonisation. The most proactive strategy would have been to test cases and contacts both before and after decolonisation therapy. However, this would have been operationally complex, logistically challenging and potentially problematic in cases where MRSA eradication was not achieved. It was decided not to test cases or contacts who were asymptomatic post-treatment.

It was reported that most children and adolescents at the gathering engaged in close contact play using the bouncy castles and other inflatable play structures, in many cases for several hours. Parents of cases recall the day in question as humid and rainy, with the bouncy castles crowded, playing in what was described as a ‘rough-and-tumble’ or physical fashion. While a formal cohort study was not possible – due to no definitive total attendee list – no other exposure appeared as plausible as the close contact play that occurred using the bouncy castles.

Moreover, the body site distribution of symptoms typically appeared consistent with the physical contact, with the bouncy castle surface and with other children and adolescents, entailed in the play at the party. Such play activities may have been conducive to breakages to the skin barrier, increasing the risk of infection inoculation. Other reported CA-MRSA outbreaks – such as among American footballers with turf burns [2] – have proposed an association with skin barrier breakage in facilitating transmission and development of clinical infection.

Consideration was given to swabbing the surface of bouncy castles and inflatable items used at the event. For several reasons, this was not pursued. Firstly, considerable time had elapsed since the event by the time swabbing was considered, and evidence as to the ex vivo survivability of MRSA is not clear. Secondly, the swabbing process would have been logistically and practically complex. Thirdly, the OCT members agreed that neither a positive nor a negative surface swab result would confirm or discount the hypothesis that fomite transmission played a role in the outbreak.

The epidemiology of this outbreak is suggestive of a point-source, with limited evidence of secondary transmission identified. It was not possible to determine the exact source or transmission dynamics. It is likely that an individual, or individuals, in attendance had symptomatic infection leading to onward transmission, which is consistent with a sufficient inoculum contributing to short observed incubation periods and high apparent attack rate. An individual with asymptomatic colonisation is also a possible, but less likely, source. It was considered that contamination of the inflatable play structures before the event was less likely due to factors including the extent of transmission at the event. This outbreak highlights the risk of CA-MRSA transmission associated with the use of bouncy castle and other inflatable play structures. Providers of such equipment should be made aware of this risk and take appropriate IPC measures to ensure that adequate decontamination practices are employed between use.

In Ireland, there is no formal surveillance system to monitor CA-MRSA cases or SSTI clusters, and detection of MRSA is not notifiable in Ireland, although *Staphylococcus aureus* bacteraemia is listed as notifiable [22]. As such, the prevalence of asymptomatic MRSA carriage and incidence of symptomatic MRSA SSTI are not known. Screening for MRSA depends on context and is typically limited to situations like between-hospital transfers and patients at high risk of severe illness, such as patients with indwelling devices and long-term debilitated patients [23]. In some countries, including Norway and Denmark, and in subnational regions, such as Connecticut and Western Australia, MRSA is notifiable [24-26]. In Ireland, MRSA surveillance and investigation is coordinated by stakeholders, including the National MRSA Reference Laboratory. Making MRSA notifiable, which would require a statutory instrument, could strengthen such work by enabling more systematic surveillance and earlier outbreak detection of this pathogen.

Finally, given the extent of the outbreak, the limited evidence of secondary transmission is notable. This suggests that a combination of prompt recognition of linked cases, early provision of clinical care, including decolonisation of cases and family contacts, along with effective communication and implementation of IPC measures by families may have successfully supported outbreak control.

This outbreak response exhibited several limitations. Firstly, a definitive attendee list was not obtainable for this gathering. This was because invitation occurred informally, through word of mouth and messaging platforms such as WhatsApp. Most attendees came from the locality and many from the same housing estate as the event hosts.

A second limitation is that the line list document, which constituted the basis for interviews with parents and guardians of cases, was prepared rapidly on the day of outbreak notification and so was not exhaustive. For example, recent overseas travel of cases or their family members was not systematically documented, which could in theory have provided insights as to the origin of this outbreak. Overseas travel has been shown on several occasions to be a means of importation of MRSA strains into Ireland [27-29].

A third limitation is that data on antimicrobial therapy – specific agent, dose and duration of treatment – were not systematically documented for all outbreak cases. This was not feasible, due to a lack of integration between health datasets at primary care, hospital and public health level during this outbreak. The issue of data fragmentation and siloes in the Irish context is well-recognised [30,31].

A final limitation is that the outbreak case definitions only pertained to symptomatic MRSA infection and not individuals asymptomatically colonised. Ideally, a ‘screen and clean’ strategy would test cases and contacts both before and after decolonisation therapy. The OCT did not pursue such a strategy, for several reasons. Firstly, the efficacy of decolonisation therapy in achieving MRSA eradication is known to be less than total. Secondly, the potential situation of individuals asymptomatically colonised long-term would have precluded outbreak closure. Finally, the logistics of repeatedly testing all cases and their contacts until achievement of negative test results would have been operationally highly complex. Nonetheless, this constitutes a limitation of our outbreak response.

## Conclusion

This was an unusual and complex community outbreak of MRSA SSTI in children and adolescents following a social event in eastern Ireland, linked to bouncy castle play. This is a novel mechanism for an outbreak of this size. Public health practitioners should be aware of the risk of CA-MRSA outbreaks in contexts involving prolonged, close physical contact, such as play activities and contact sport. Multidisciplinary governance and collaboration were essential to the outbreak investigation and response.

## Data Availability

The individual-level dataset analysed in this study cannot be shared publicly due to medicolegal restrictions regarding patient data confidentiality. Investigation and control of infectious disease in Ireland is a statutory mandate of the medical officer of health, under the 1947 and 2007 Health Acts and the 1981 Infectious Disease Regulations [32–34]. Aggregated data are available on a case-by-case basis upon reasonable request to the article authors.

## Supplementary Data

794000 Supplementary Material
