# Supplementary material for: An outbreak of Panton-Valentine leucocidin-producing meticillin-resistant Staphylococcus aureus skin and soft tissue infection associated with bouncy castle use, Ireland, 2025
Source: Euro Surveill. 2026 Sep 10;31(36):2500975. doi: 10.2807/1560-7917.ES.2026.31.36.2500975 (PMC13570234; doi:10.2807/1560-7917.ES.2026.31.36.2500975)
Supplement: Supplementary file 1 [file 25-00975_McGLACKEN-BYRNE_Supplement.pdf]

Supplementary File – Information Issued to Cases' Families

This supplementary material is hosted by Eurosurveillance as supporting information alongside the article *An outbreak of Panton-Valentine leucocidin-producing meticillin-resistant Staphylococcus aureus skin and soft tissue infection associated with bouncy castle use, Ireland, 2025*, on behalf of the authors, who remain responsible for the accuracy and appropriateness of the content. The same standards for ethics, copyright, attributions and permissions as for the article apply. Supplements are not edited by Eurosurveillance and the journal is not responsible for the maintenance of any links or email addresses provided therein.

This leaflet provides guidance for managing infectious soft tissue infections and limiting spread within the household. Always follow the advice of your doctor. Written by the Dept. of Paediatric Emergency Medicine in [name of hospital] and updated by the Department of Public Health following an Outbreak Control Team meeting on the [date of meeting].

The Patient with Symptoms

Treatment Plan

1. **Antibiotics** – Take exactly as prescribed by your doctor.
2. **Contamination reduction** – For 5 days:
    - Over the counter **Octenisan® for < 1 year and Hibiscrub® for > 1 year old** body wash once daily for 5 days
    - **Nasal antibiotic ointment** (e.g. mupirocin) for 5 days
3. **Exclusion Advice**
    - The patient should not attend school or crèche until such time as acute infection symptoms have fully resolved. Once symptoms have resolved the child can return to school whilst simultaneously completing their contamination reduction plan for 5 days.

Patients should not attend swimming pools, gyms, sports clubs training or games until such time as symptoms have fully resolved.

Do

- ✓ **stay at home if you are symptomatic**
- ✓ **do launder bedclothes, towels, linen and used clothes before starting decolonising treatment**
- ✓ **complete the full treatment plan**
- ✓ **keep sores, blisters and crusty patches clean and dry**
- ✓ **cover them with loose clothing or gauze bandages**
- ✓ **wash your hands frequently**
- ✓ **wash your face cloths, sheets and towels at a high temperature**
- ✓ **wash or wipe down toys with soap and warm water**

Don't

- × **do not touch or scratch sores, blisters or crusty patches - this also helps stop scarring**
- × **do not have close contact with children, or people with diabetes or a weakened immune system**
- × **do not share face cloths, sheets or towels**
- × **do not prepare food for other people**
- × **do not go to the gym**
- × **do not play contact sports**

If symptoms do not improve, worsen, or new symptoms develop, contact your doctor immediately

For Family Members (No Symptoms)

Routine contamination reduction plans are not usually required for family members or household contacts. Hand hygiene, regular cleaning and disinfection of shared areas in the house and avoiding sharing personal items e.g. towels, face cloths and linen, in a household contribute to reducing the risk of transmission of infection in the household.

Activities

Asymptomatic family members can continue their normal daily activities.

Maintain **good hand hygiene** and household hygiene practices.

General Hygiene Advice

Wash hands frequently with soap and water.

Do not share towels, bedding, or clothing during treatment.

Wash clothes, bed linen, and towels regularly.

Keep nails short and clean.

Do

- ✓ **stay at home if you become symptomatic otherwise carry on as normal**
- ✓ **wash your hands frequently**
- ✓ **wash your face cloths, sheets and towels regularly**
- ✓ **wash or wipe down toys with soap and warm water**

Don't

- × **do not share face cloths, sheets or towels until treatment plan(s) are complete**

If symptoms develop, contact your doctor.
